# The Role of Integrative Taxonomy in the Conservation Management of Cryptic Species: The Taxonomic Status of Endangered Earless Dragons (Agamidae: *Tympanocryptis*) in the Grasslands of Queensland, Australia

**DOI:** 10.1371/journal.pone.0101847

**Published:** 2014-07-30

**Authors:** Jane Melville, Katie Smith, Rod Hobson, Sumitha Hunjan, Luke Shoo

**Affiliations:** 1 Department of Sciences, Museum Victoria, Melbourne, Victoria, Australia; 2 Queensland Parks & Wildlife Service, Toowoomba, Queensland, Australia; 3 School of Biological Sciences, University of Queensland, St Lucia, Queensland, Australia; Institute of Biochemistry and Biology, Germany

## Abstract

Molecular phylogenetics is increasingly highlighting the prevalence of cryptic species, where morphologically similar organisms have long independent evolutionary histories. When such cryptic species are known to be declining in numbers and are at risk of extinction due to a range of threatening processes, the disjunction between molecular systematics research and conservation policy becomes a significant problem. We investigate the taxonomic status of *Tympanocryptis* populations in Queensland, which have previously been assigned to *T. tetraporophora*, using three species delimitation approaches. The taxonomic uncertainties in this species-group are of particular importance in the Darling Downs Earless Dragon (*T. cf. tetraporophora*), which is ranked as an endangered ‘species’ of high priority for conservation by the Queensland Department of Environment and Heritage Protection. We undertook a morphological study, integrated with a comprehensive genetic study and species delimitation analyses, to investigate the species status of populations in the region. Phylogenetic analyses of two gene regions (mtDNA: *ND2*; nuclear: *RAG1*) revealed high levels of genetic divergence between populations, indicating isolation over long evolutionary time frames, and strongly supporting two independent evolutionary lineages in southeastern Queensland, from the Darling Downs, and a third in the Gulf Region of northern Queensland. Of the three species delimitation protocols used, we found integrative taxonomy the most applicable to this cryptic species complex. Our study demonstrates the utility of integrative taxonomy as a species delimitation approach in cryptic complexes of species with conservation significance, where limited numbers of specimens are available.

## Introduction

The increasing use of molecular phylogenetic techniques has highlighted the prevalence of cryptic species, which are morphologically similar organisms with long independent evolutionary histories [1], [2]. Cryptic species, like other species, may become at risk of extinction due to a range of threatening processes. Because of this, it is important that molecular systematic research and a taxonomic framework for conservation are closely aligned. Currently, most molecular systematics studies identifying cryptic species do not incorporate formal taxonomic revisions [3], which causes a disjunction with conservation management authorities that require taxonomic recognition of species or at least subspecies. For example, in the state of Queensland, Australia, under the *Nature Conservation Act 1992* there is a basic requirement of taxonomic recognition to nominate a species to qualify for threatened status. Thus, there is a need for greater integration between taxonomy, molecular systematics and conservation management.

For species of conservation concern there are often few specimens available for taxonomic assessment, making taxonomic revisions using traditional methods difficult. This is where recent species delimitation methods [3], [4], [5], incorporating multiple lines of evidence (e.g., field ecology, molecular systematics and morphology), become of particular importance in the management of biodiversity. A case in point is the taxonomic status of grassland Earless Dragons in Queensland (*Tympanocryptis* spp.). The genus *Tympanocryptis* currently comprises eight recognised species of small ground-dwelling dragons. *Tympanocryptis* is distinguished by a tympanum covered by scales and the loss of a phalange in the fifth toe of the rear foot [6]. Although the genus is well characterised there remain taxonomic problems with several species groups and one that has received relatively little taxonomic attention is *T. tetraporophora*.

*Tympanocryptis tetraporophora* Lucas and Frost 1895 was described from specimens collected on the Horn Expedition into central Australia in 1894. Although Lucas and Frost noted colour variations between the three *T. tetraporophora* specimens examined, specimens were distinguished from *T. lineata* by the presence of two femoral pores and two preanal pores. Subsequently, in 1948 Mitchell [7] detailed additional characters that distinguish *T. tetraporophora*: (a) a very elongate form; (b) a constant position for the nostril, having a greater number of scales separating it from the upper labials than in *T. lineata*; and (c) a comparatively constant and definite tubercule shape. Mitchell [7] also recognised considerable variation in colouration, both between and within populations of *T. tetraporophora*. Beyond such observations, there has been little consideration of the taxonomic relevance of variation within *T. tetraporophora*, despite its vast distributional range and the diversity of habitats in which it occurs.

**Figure 1:**
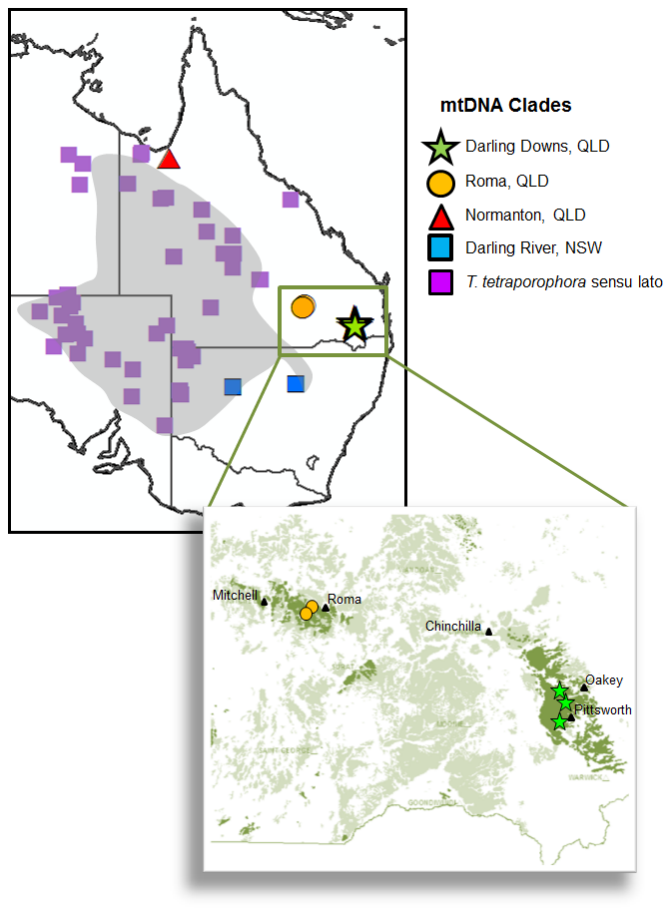
Distribution of the samples sequenced in the current study for *Tympanocryptis tetraporophora*. Symbols represent different mtDNA clades identified in Fig. 2 and shading represents the current distribution of *Tympanocryptis tetraporophora* [22]. Map inset shows the Darling Downs, with locations of samples included in our study and the distribution of pre-European (1750) vegetation: tussock grasslands (dark green); brigalow/belah and *Callitris* forest (light green). Plotted using ArcGIS 10.0 onto “Countries 2006” and NVIS Major Vegetation Groups (Version 4.1) - PRE 1750.

*Tympanocryptis tetraporophora* occurs across approximately a quarter of continental Australia, including South Australia, the Northern Territory, Queensland and New South Wales (Fig. 1). This species occupies an impressive climatic gradient, ranging from the arid interior of South Australia, to semi-arid New South Wales and into the tropical grasslands of the Gulf region in northern Queensland. Additionally, it occurs in a variety of habitats, such as stony desert plains, inland floodplains, black soil plains and tropical savannah grasslands. Recently, it has also been recognised that there is an isolated population in the Darling Downs of south-eastern Queensland, which has tentatively been assigned to *T. teraporophora* [8].

The *Tympanocryptis* populations from the Darling Downs were originally assigned to the endangered grassland earless dragon, *T. pinguicolla* [9]. This species was discovered living in crop-lands and remnant Bluegrass grasslands of south-eastern Queensland, which is one of the most endangered ecosystems in the state [10]. More recently it has been shown that these populations of Earless Dragons are unrelated to *T. pinguicolla*, based on morphological and genetic data, and are more closely related to *T. tetraporophora* [8]. Currently, the Darling Downs Earless Dragon is listed as *T. cf. tetraporophora* by the Queensland Department of Environment and Heritage Protection and is a ranked as an endangered species of high priority for conservation. Thus, research on variation within *T. tetraporophora* is of particular importance to resolve the issue of the taxonomic status of populations in south-eastern Queensland.

We present an integrated taxonomic study of *Tympanocryptis tetraporophora* sensu lato to examine diversification in this species complex. We sequenced two gene regions, incorporating mtDNA (ND2) and nuclear (RAG1) genes, and undertook extensive phylogenetic, morphological, species delimitation analyses and species descriptions. Our study not only contributes valuable information to resolving taxonomic issues in this group but also provides a case study of how integrative taxonomy protocols can be incorporated into the conservation management of cryptic species.

## Methods

### Tissue samples

To provide phylogenetic context of *T. tetraporophora*-like populations from Queensland, we endeavored to maximize geographic spread of samples from throughout the species' range (Fig. 1). For the current study we used tissue samples from museum collections, giving a total of 100 new samples sequenced for this study. Lists of all museum tissue samples used for molecular work in this study, as well as locality data and GENBANK accession numbers, are included in Table S1. In addition, we included previously published mtDNA sequence [11] from the ethanol-fixed *T. tetraporophora* lectotype (MNV D7701), a specimen collected in 1894 from the Adminga and Dalhousie region of South Australia. An additional thirty-four previously published DNA sequences were also incorporated in phylogenetic analyses.

### DNA sequencing and alignment

Genomic DNA was isolated from liver samples using Proteinase K digestion and chloroform-isoamyl alcohol extraction or using a DNAeasy tissue extraction kit (Qiagen) as per manufacturer's instructions. For all specimens, a fragment (∼1200 bp) of the mtDNA genome was targeted that includes the entire protein-coding gene *ND2* (NADH dehydrogenase subunit two) and flanking genes encoding tRNA^Trp^, tRNA^Ala^, tRNA^Asn^, tRNA^Cys^, tRNA^Tyr^. For a subset of specimens, selected to encompass all mtDNA lineages, we sequenced a ∼1200 bp of recombination activating gene-1 (*RAG1*) exon in the N-terminal domain [12]. Oligonucleotide primer pairs used in PCR amplification and sequencing of mitochondrial and nuclear genes are detailed in [13]. Amplifications were performed in 25 µl volumes in the presence of 1.5 mM MgCl_2_, 0.2 mM dNTPs, 0.2 µM of forward and reverse primer, 1x Qiagen PCR buffer and 1 Unit of HotStarTaq DNA polymerase (Qiagen). Thermal cycling conditions consisted of an initial denaturing and enzyme activation step at 95°C for 15 minutes followed by 40 cycles of denaturing at 95°C for 20 seconds, annealing at 48°C (*RAG1*) or 55°C (*ND2*) for 20 seconds, and extension at 72°C for 90 seconds, using a Corbett thermocycler. Negative controls were run for all amplifications. PCR amplifications were visualised on a 1.2% agarose mini-gel or using a QIAxcel gel analysis system. Amplified products were purified using GFX spin columns or using SureClean Plus (BIOLINE). Purified product was sent to Macrogen (Korea) for sequencing.

Sequence chromatograms were edited using Geneious 6.1.2 (Biomatters Ltd.) to produce a single continuous sequence for each specimen. Mitochondrial DNA sequences were aligned and protein-coding regions were translated to amino acids to check alignment and for stop codons.

### Phylogenetic analyses

Phylogenetic analyses of all samples for the mtDNA gene region and a subset of specimens for the RAG1 gene region were undertaken using a Bayesian framework in MrBayes 3.2 [14], with a partitioned GTR+I+Γ model [15] implemented for both datasets and model parameter values estimated from the data. We assumed three (codon positions 1–3) partitions for the protein-coding mtDNA and RAG1 genes and a fourth partition for the mtDNA tRNAs regions included. This model was chosen based on preliminary analyses using MrModeltest 2. 3 [16], with the Akaike Information Criterion. Four Markov chains were used in each of two simultaneous runs starting from different random trees. The analyses were run for 10 million generations and the standard deviation of split frequencies were used as a convergence diagnostic to confirm suitability of run lengths. It was confirmed that potential scale reduction factor (PSRF) values were close to 1.0, indicating that an adequate sample of the posterior probability distribution had been achieved [14]. In addition, the outputs were examined using Tracer v1.3 [17] to check that stationarity had been reached.

We used a Bayesian framework for species tree estimation, incorporating both gene regions (*ND2* and *RAG1*), to determine whether the suggested new species constitute evolutionary lineages across the two gene regions. To do this analysis we used only samples that contained sequence data for both gene regions, resulting in two datasets of 45 individuals. We used *BEAST, enabled in BEAST v1.7.5, to co-estimate the two gene trees embedded in a shared species tree [18]. Unlinked substitutions models were employed across the loci and GTR+I+Γ models of sequence evolution were implemented. These models were chosen based on preliminary analyses MrModeltest 2. 3 [16], with the Akaike Information Criterion. A Yule process species tree prior was specified and the gene tree priors were automatically specified by the multispecies coalescent. The analysis was run for 50 million generations. The output was examined using Tracer v1.3 [17] to check that stationarity had been reached. An estimate of the species tree was obtained using TreeAnnotater.

### Morphology

Specimens were examined from across the range of *T. tetraporophora* from the collections of the South Australian Museum, Adelaide (SAMA), the Queensland Museum (QM), the Australian Museum (AM) and Museum Victoria (NMV). A list of all voucher specimens used for morphological analyses is provided in Appendix S1. Type specimens examined were from Museum Victoria (*T. tetraporophora*) and the Australian Museum (*T. karumba* Wells and Wellington 1985). Specimens relating to the mtDNA clade from the Darling River in NSW were not available for the current study, thus, this clade was not included in analyses. Thirteen meristic and metric characters (Table 1), which were thought to be potentially diagnostic, were recorded. Electronic callipers were used for all morphological measures to the nearest 0.1 mm and all bilateral counts and measurements were recorded on the left side only.

**Table 1:** **Morphological measurements and meristic character counts for four genetic lineages identified in species tree analyses (** **Fig. 3** **).**

	*T. tetraporophora* sensu stricto N = 162	Roma-area N = 2	Darling Downs N = 7	Normanton-area N = 3
SVL	53.6 (±0.47)	46.9 (±6.84)	53.9 (±3.44)	52.3 (±3.61)
HL	17.7 (±0.13)	16.3 (±3.60)	17.7 (±0.78)	17.5 (±0.56)
HW	12.4 (±0.10)	11.8 (±1.36)	12.1 (±0.56)	12.3 (±0.67)
HD	8.1 (±0.07)	7.7 (±1.65)	8.4 (±0.31)	8.0 (±0.36)
SL	13.3 (±0.09)	11.0 (±0.00)	12.1 (±0.46)	10.0 (±0.58)
IL	13.8 (±0.11)	12.0 (±0.00)	11.3 (±0.29)	11.3 (±0.67)
IN	7.7 (±0.07)	8.5 (±0.50)	9.1 (±0.26)	6.0 (±0.0)
NS	5.2 (±0.05)	4.0 (±0.00)	4.7 (±0.18)	4.7 (±0.33)
SO	12.0 (±0.08)	11.0 (±1.00)	11.6 (±0.30)	12.0 (±0.00)
AG	30.9 (±0.34)	27.6 (±3.05)	26.6 (±0.168)	28.8 (±2.28)
LegL	37.7 (±0.31)	34.6 (±5.72)	38.0 (±1.97)	37.3 (±2.24)
SLam	17.3 (±0.10)	18.5 (±0.50)	18.0 (±0.31)	16.3 (±0.67)
TailL	82.9 (±0.89)	63.14	74.9 (±5.28)	84.1 (±6.22)
PP	2.0 (±0.01)	2.00 (±0.00)	1.9 (±0.14)	2.0 (±0.00)
FP	2.0 (±0.02)	0.0 (±0.00)	0.0 (±0.00)	2.0 (±0.00)

*T. tetraporophora* sensu stricto; Roma-area; Darling Downs; and Normanton-area. All measurements are in mm and mean (± SE) are provided. Abbreviations are: snout-vent length (SVL), head length (HL), head width (HW), head depth (HD), axilla – groin length (AG), hind limb length (LL), number of inter-nasals (IN), number of infralabials (IL), number of supralabials (SL), number of scales between nasal and supralabial (NS), number of sub-digital lamellae on longest toe (SLAM), number femoral pores (FP); number of pre-anal pores (PP), tail length (TailL), and number of sub-ocular scales (SO).

We then performed a discriminant function analysis (DFA) to assess how well putative species could be classified based on morphology. Two variables were excluded from the DFA: preanal pores (PP), which was invariant, and tail length (TailL), which contained a high number of broken tails. Prior to analysis, body size (represented by snout-vent length) was removed from all metric variables by regressing snout-vent length against the variable and using the residuals in the DFA. Analysis of variance (ANOVA) was used to determine if there was a significant difference in univariate variables between the sexes. Based on these results DFAs were conducted separately on males and females.

### Species Delimitation Assessment

We selected three methods of species delimitation (see [5] for a comparison of methods): Mitochondrial Tree - Morphological Character Congruence; the Wiens and Penkrot protocol [4]; and Integrative Taxonomy. We selected these approaches because they employ data typically gathered in taxonomic revisions, with limited sampling of specimens and populations, and represent the current methods that combine different data and different lines of evidence in an integrative way [5].

The Mitochondrial Tree – Morphological Character Congruence (MTMC) approach has been formalized by Miralles and Vences [5] and represents the most common practice in zootaxonomic studies combining evidence from DNA sequences and morphological data. We used this method to define species as morphologically diagnosable units, through fixed and unambiguous morphological character states, that are revealed by a mtDNA tree.

The Integrative Taxonomic (ITAX) approach follows the principle that as many lines of evidence as available should be combined to delimit species [5], such as genetic (mtDNA and nuclear), morphological, distributional and ecological data [4]. We modified the criteria for assessment outlined in Miralles and Vences [5], where they specify criteria incorporating both sympatric and not-necessarily sympatric putative species. We excluded the criteria for sympatric species in our methodology, as none of the putative species in *T. tetraporophora* sensu lato are known to occur sympatrically.

The protocol developed by Wiens and Penkrot (WP) [4] to delimit species uses an approach based on nonrecombining molecular phylogenetic data. This approach compares the phylogenetic pattern of specimens assigned to a focal species, relative to other closely related species. Wiens and Penkrot [4] provide a flow chart protocol that leads to alternative species-level decisions, specifying that gene flow between populations indicates non-exclusive lineages. We used this method to test the status of each nominal species in the mtDNA species tree, integrating information from the nuclear data set to assess for evidence of gene flow between lineages.

### Nomenclatural Acts

The electronic edition of this article conforms to the requirements of the amended International Code of Zoological Nomenclature, and hence the new names contained herein are available under that Code from the electronic edition of this article. This published work and the nomenclatural acts it contains have been registered in ZooBank, the online registration system for the ICZN. The ZooBank LSIDs (Life Science Identifiers) can be resolved and the associated information viewed through any standard web browser by appending the LSID to the prefix “http://zoobank.org/”. The LSID for this publication is: urn:lsid:zoobank.org:pub: FFF5C4BE-1C46-4C78-99C5-8958210B66D1. The electronic edition of this work was published in a journal with an ISSN, and has been archived and is available from the following digital repositories: PubMed Central, LOCKSS.

## Results

### Mitochondrial DNA phylogenetics

To investigate phylogenetic relationships of *Tympanocryptis tetraporophora* sensu lato, 100 new sequences and 34 previously published sequences were analyzed for the *ND2* protein coding gene. The alignment comprised 1410 characters: 755 characters were variable and 567 characters were parsimony informative. A GTR+I+Γ model was selected as the best fitting model for likelihood analysis using AIC criteria. Model parameters were: gamma  = 0.789; proportion of invariable sites  = 0.282; substitution rates A↔C 0.5364, A↔G 12.2216, A↔T 0.6706, C↔G 0.4734, C↔T 6.2535, G↔T 1.0000; and, nucleotide frequencies A = 0.3778, C = 0.3263, G = 0.0801 and T = 0.2158.

**Figure 2:**
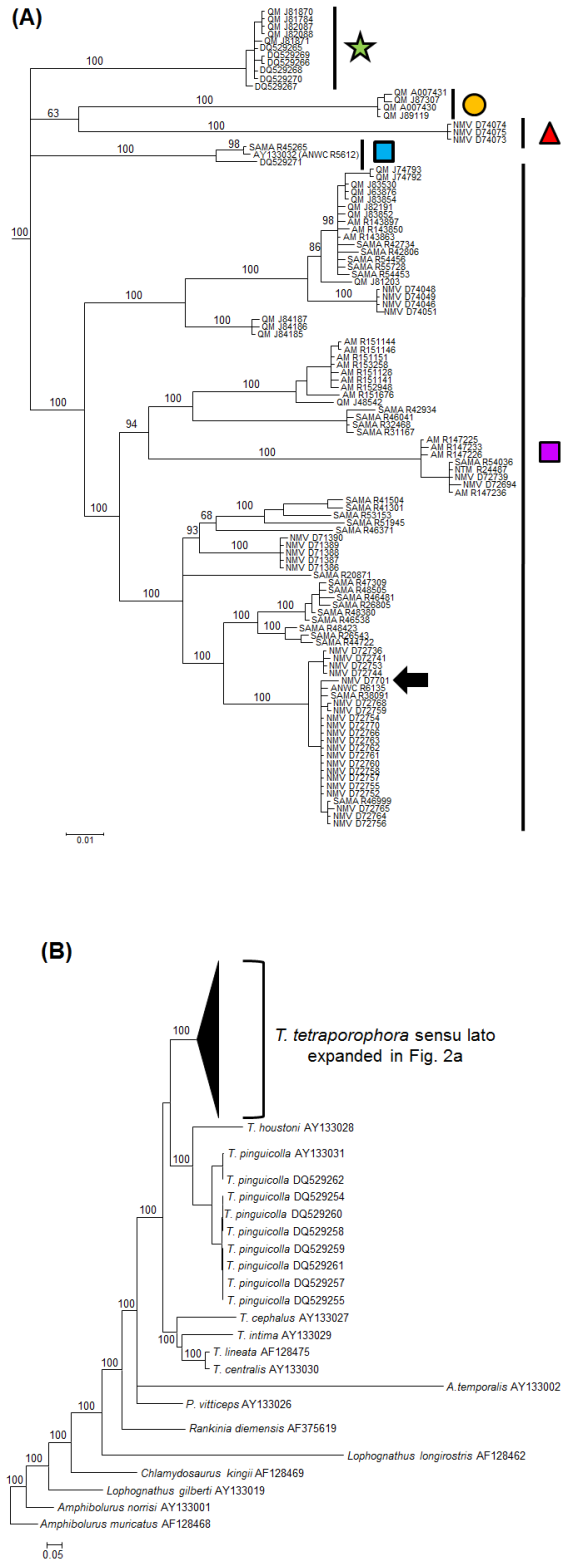
Bayesian phylogenetic tree for *Tympanocryptis tetraporophora* based on ∼1400 bp mitochondrial DNA (*ND2*). Samples sequenced in the current study are designated by museum registration numbers and previously published sequences are designated by GENBANK numbers. Figure (A) represents ingroup samples and (B) outgroups. Posterior probabilities are provided on the branches. Symbols on the clades are those from Fig. 1. Large arrow indicated the phylogenetic position of the *T. tetraporophora* lectotype (NMVD7701).

The mtDNA Bayesian phylogeny (mean ln-likelihood −12825.59) strongly supported the *Tympanocryptis tetraporophora* complex as an evolutionary lineage (Fig. 2B). Within this clade there are five well supported monophyletic lineages corresponding to geographic regions (Fig. 1, Fig. 2A): 1. *T. tetraporophora* sensu stricto from inland QLD, NSW, SA and NT; 2. the Darling River in northern NSW; 3. Roma-area, QLD; 4. Darling Downs, QLD; and 5. Normanton-area, QLD. The phylogenetic relationships between these clades is unresolved. There is significant phylogeographic structure within *T. tetraporophora* sensu stricto clade, with a basal lineage from northern Queensland that is the sister clade to two well supported monophyletic lineages, one occurring in southwestern QLD, western NSW and eastern NT, and a second lineage occurring in north-eastern SA and southern NT. Mean uncorrected genetic distance between the five lineages of the *T. tetraporphora* complex ranged from 6.3% to 8.7% (Table 2). Based on previous age estimates using fossil-calibrated relaxed molecular clock analysis [13], these five lineages date back to the late Miocene or Pliocene.

**Table 2:** **Uncorrected genetic distance between five well supported mtDNA lineages.**

	Clade 1	Clade 2	Clade 3	Clade 4
Clade 2	6.4% (5.7–7.7)	-		
Clade 3	7.6% (7.1–8.6)	7.5% (7.4–7.7)	-	
Clade 4	7.0% (6.4–8.2)	6.3% (6.1–6.4)	7.2% (7.1–7.4)	-
Clade 5	8.3% (7.5–9.4)	8.2% (8.1–8.3)	8.7% (8.6–8.9)	8.2% (8.0–8.4)

The five clades are (Fig. 1): 1. *T. tetraporophora* sensu stricto from inland QLD, NSW, SA and NT; 2. the Darling River in northern NSW; 3. Roma-area, QLD; 4. Darling Downs, QLD; and 5. Normanton-area, QLD. Values given are mean uncorrected genetic distance (min - max).

### Nuclear DNA phylogenetics

**Figure 3:**
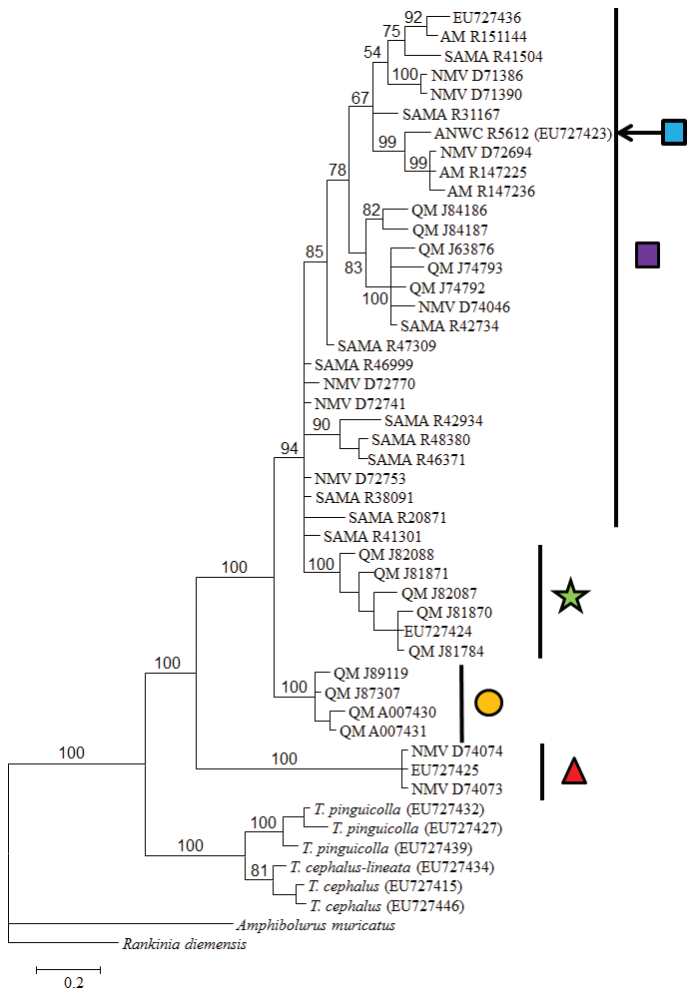
Bayesian phylogenetic tree for *Tympanocryptis tetraporophora* based on ∼1200 bp nuclear DNA (*RAG1*). Samples sequenced in the current study are designated by museum registration numbers and previously published sequences are designated by GENBANK numbers.

Thirty-seven new sequences and twelve previously published sequences were analyzed for the *RAG1* protein coding gene. The alignment comprised 1226 characters: 124 characters were variable and 82 characters were parsimony informative. A GTR+I+Γ model was selected as the best fitting model for likelihood analysis using AIC criteria. Model parameters were: gamma  = 0.945; proportion of invariable sites  = 0.731; substitution rates A↔C 1.0690, A↔G 5.0531, A↔T 0.4131, C↔G 0.4545, C↔T 7.2461, G↔T 1.0000; and, nucleotide frequencies A = 0.3249, C = 0.2196, G = 0.2191 and T = 0.2364. The Bayesian tree (Fig. 3) was largely consistent with that obtained from mitochondrial data (mean ln-likelihood −3045.45). Three of the monophyletic lineages recovered in mtDNA were also well supported (100% posterior probability) with the nuclear dataset: Roma-area, QLD (Clade 3); Darling Downs, QLD (Clade 4); and Normanton-area, QLD (Clade 5). However, the phylogenetic relationships between the Darling Downs, QLD, Clade 5 and *T. tetraporophora* sensu stricto (Clade 1) lacked the resolution recovered using mtDNA. The Darling River in northern NSW clade (Clade 2) recovered in the mtDNA was not supported in the nuclear DNA, where it falls within the main *T. tetraporophora* sensu stricto clade from inland QLD, NSW, SA and NT (Clade 1).

### Estimate of species tree

Two datasets (mtDNA & nDNA) incorporating the same 45 individuals were used in a species tree analysis. A new MrModeltest 2. 3 analysis conducted on the reduced mtDNA dataset selected an HKY+I+Γ model as the best fitting model using AIC criteria. Model parameters were: gamma  = 0.8979; proportion of invariable sites  = 0.7472; TRatio  = 4.3400; and, nucleotide frequencies A = 0.3221, C = 0.2221, G = 0.2168 and T = 0.2390.

**Figure 4:**
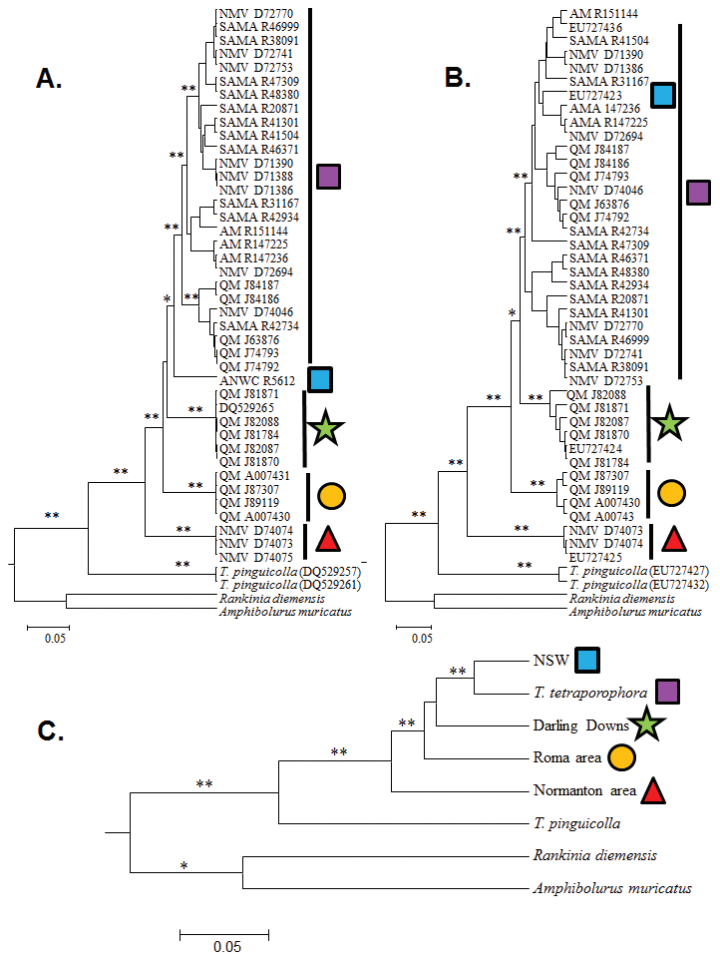
Gene and species tree phylogenies based on datasets inferred using *Beast: A). mtDNA (*ND2*); B). nuclear (*RAG1*); and C). species tree. Clade posterior probabilities are indicated on branches (*>90%; **>98%). Symbols on the clades are those from Fig. 1.

The posterior parameter value estimates from the species delimitation analysis (*BEAST) were characterised by high (>200) effective sample sizes and convergence of the individual runs was confirmed from assessments using Tracer. The maximum clade credibility trees from the posterior sets of species trees inferred from both genes were similar (Fig. 4). The topology of the mtDNA tree was the same as that for the full dataset. In the topology of the *RAG1* tree, however, one sample that comprises a well-supported lineage in the mtDNA (Clade 2: the Darling River in northern NSW) falls within the main *T. tetraporophora* sensu stricto clade from inland QLD, NSW, SA and NT (Clade 1). Other than this discrepancy, all remaining mtDNA lineages (Clades 3, 4 & 5) are well supported (i.e.>95%) as monophyletic in the *RAG1* tree. In both gene trees, Clade 5 (Normanton-area, QLD) is strongly supported as the basal lineage to the remainder of the *T. tetraporophora* sensu lato clades. Most nodes in the species tree inferred using both *ND2* and *RAG1* gene regions were well supported (Bayesian posterior probability >95%), with the exception of Clade 3 (Roma-area) being the sister species to Clades 1, 2 & 4 (53.5%).

### Morphology

Significant differences were identified between male and female *T. tetraporophora* senus stricto for relative head length (F_1,172_ = 35.766, P<0.001), relative axilla – groin length (F_1,172_ = 17.621, P<0.001), and relative hind-leg length (F_1,172_ = 58.080, P<0.001). Consequently, subsequent discriminant function analyses (DFA) were conducted separately on males and females.

**Figure 5:**
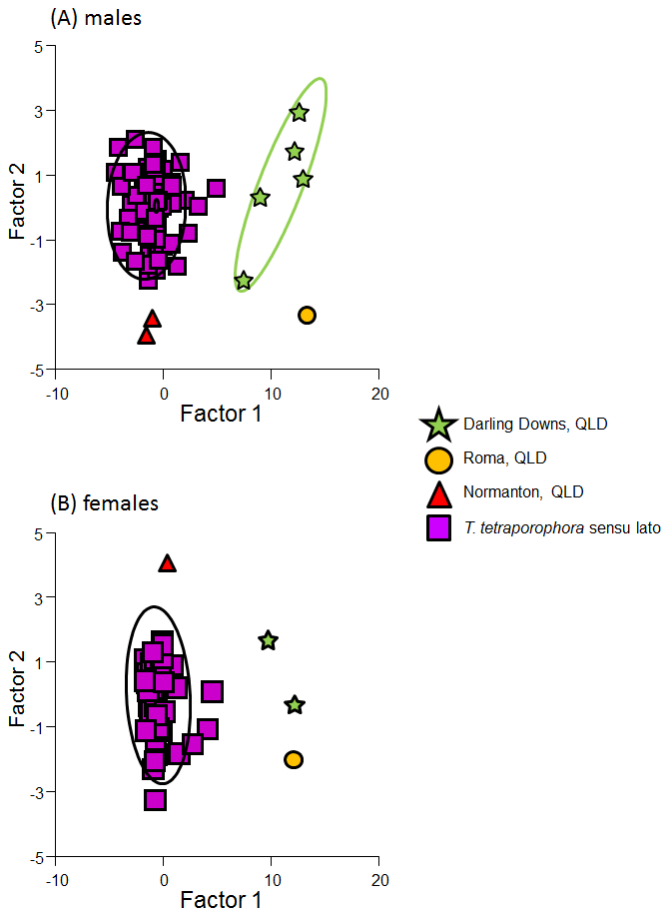
Plot of canonical variables 1 and 2 from the discriminant function analysis of morphology showing the relative position for each of the four putative species for (A) males and (B) females. Ellipses represent 95% confidence ellipses for samples sizes >2.

The four phylogenetic clades identified in the species tree analysis could be distinguished from each other in the discriminant functional analysis of both males (Wilks' λ_13, 3, 105_ = 0.052, F_39,262_ = 12.42, P<0.001) and females (Wilks' λ_13, 2, 62_ = 0.162, F_26,100_ = 5.709, P<0.001). DFA correctly classified 98% of males into the putative species, where one Normanton-area (Clade 5) animal was misclassified as *T. tetraporophora* sensu stricto (Clade 1) and one Roma-area (Clade 3) animal was misclassified as a Darling Downs (Clade 4) animal. In females, 100% of animals were correctly classified into the putative species. A plot of canonical scores (CV1 & CV2) from the discriminant function analysis shows the relative position of each putative species for both males and females (Fig. 5). The first canonical variable (CV1) was found to account for 93.1% of dispersion in males and 93.4% of dispersion in females. In males CV1 discriminated putative species based on number of femoral pores (correlation between this variable and CV1 = −0.96), while CV2 was mainly based on number of supralabials (correlation  = 0.89). In females, CV1 discriminated putative species based on number of femoral pores (correlation  = −0.91) and the number of infralabials (correlation  = −0.59), while CV2 was based on the number of inter-nasals (correlation  = −0.62).

### Species Delimitation Assessment

#### The Mitochondrial Tree – Morphological Character Congruence (MTMC)

**Figure 6:**
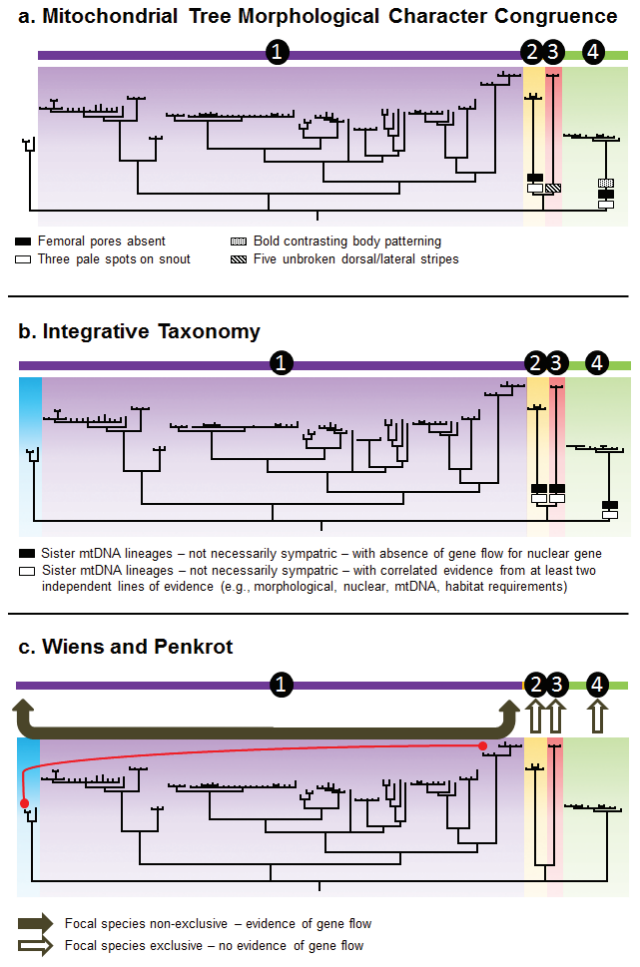
Results from the Mitochondrial Tree – Morphological Character Congruence (MTMC), Integrative Taxonomic (ITAX) and Wiens and Penkrot (WP) approaches to species delimitation. Taxonomies are summarized above each figure by a horizontal multicolored bar, each segment representing a different species. Red line in WP approach represent evidence of gene flow between lineages. Figure template adapted from [5].

All available specimens were separated into groups based on the independent lineages supported in the mtDNA tree and then assessed for fixed and unambiguous morphological character states (Fig. 6). We were unable to include populations from the Darling River in NSW in this assessment protocol, as specimens were not available for our study. We identified four morphologically diagnosable lineages that were also resolved as independent lineages in the mtDNA tree. Using this species delimitation method, four lineages would be considered as distinct species, which are the populations from: 1. *T. tetraporophora* sensu stricto from inland QLD, NSW, SA and NT; 2. Roma-area, QLD; 3. Normanton-area, QLD; and 4. Darling Downs, QLD.

#### The Integrative Taxonomic (ITAX)

Using the two taxonomic assessment criteria we modified from Miralles and Vences [5], we identified four distinct species using the ITAX protocol (Fig. 6). This assessment included the Darling River (NSW) mtDNA lineage, which did not have two data sources supporting species-status. In fact, the nuclear DNA provided evidence that this NSW population was actually nested within the *T. tetraporophora* sensu stricto. Consequently, this protocol identified the same four species as the MTMC approach.

#### Wiens and Penkrot (WP)

Testing the status of each nominal species in the mtDNA species tree, integrating information from the nuclear data set, we identified four species (Fig. 6), which correspond to those identified using the MTMC and ITAX approaches. We included the Darling River (NSW) mtDNA lineage in the WP protocol and identified evidence of gene flow between this lineage and *T. tetraporophora* sensu stricto using the nuclear data. For this reason, the Darling River (NSW) population was not considered to be an exclusive species using the WP protocol.

## Discussion

### Integrative taxonomy

Our study, incorporating both mitochondrial and nuclear gene regions and morphology, shows that there is far greater diversity in *Tympanocryptis tetraporophora* sensu lato than is presently recognized. Using three species delimitation protocols our work provides strong evidence of four distinct species, with all protocols giving the same results. These data provide compelling support for a taxonomic revision of the species complex with the description of three new species (refer to Systematics section below). A fifth lineage from the Darling River in northern NSW was only recovered in the mtDNA and may represent mitochondrial introgression or incomplete lineage sorting in the nDNA. These NSW samples occur along the Darling River, which is sourced from south-eastern Queensland. Thus, this mtDNA lineage may represent a past movement of animals from south-eastern Queensland down the river system, possibly through flooding events, and subsequent interbreeding with other NSW populations. Further work is required on these NSW populations to determine their evolutionary origins and taxonomic status.

The current study demonstrates the importance of integrating phylogenetic information from multiple independent loci into the taxonomic assessment of morphologically conserved groups. Our approach highlights the problems of having a limited number of specimens when using morphological approaches to taxonomy. Despite extensive examination of specimens, we found relatively few unambiguous and consistent diagnostic characters between genetic lineages and multivariate analyses found extensive overlap between independent evolutionary lineages. The results from the MTMC protocol of species delimitation based the support for a new species from the Normanton area on only one character and separated the two putative species within the Darling Downs by only one character. When these small numbers of characters are scored on only two or three specimens, the concept of them being consistent diagnostic characters is tenuous. Thus, the supporting evidence of genetics is crucial. The WP method, which was originally based just on mtDNA phylogenies [4], runs the risk of overestimating species diversity if nuclear genes are not taken into consideration. If we had not taken nuclear data into consideration when implementing WP, which identified possible gene flow between populations in NSW, we would have determined that there were five species within *Tympanocryptis tetraporophora* sensu lato. Thus, we argue that the ITAX species delimitation approach provides the greatest level of confidence in selecting the true number of species, as it is based on multiple avenues of independent data. Our study provides a foundation for future research on the earless dragons in Queensland, and in particular the Darling Downs region, by defining a set of morphological and genetic markers that allow the confident identification of species.

### Conservation implications in south-eastern Queensland

The grassland earless dragons of south-eastern Queensland have long been of conservation concern. Originally the earless dragons from the Condamine catchment, in the eastern Darling Downs (Fig. 1), were identified as *Tympanocryptis pinguicolla*, after being first discovered in the region over 30 years ago [19]. However, subsequent surveys failed to detect these dragons and they were believed to be locally extinct until their rediscovery in 2001 when a specimen was found in a grass verge along the margin of a fallow paddock [10]. It was found that these earless dragons were restricted to mixed cropping land (maize, cotton, sorghum, sunflower etc.), remnant native grasslands and grassy verges along roads (Hobson pers. com.; [10]). Based on these data, the *T. pinguicolla* populations from the Darling Downs were listed as an endangered species of high priority in Queensland. Since then the taxonomic designation of these populations has been changed to *T. cf tetraporophora*, based on phylogenetic and morphological data [8].

Our study uncovered greater diversity within the Darling Downs region of south-eastern Queensland than is currently recognized. We found strong genetic evidence (mtDNA and nuclear) for an eastern and western species of *Tympanocryptis* (Fig. 1). Based on the small number of specimens currently available for the western species from near Roma, there is very little morphological difference between the two lineages, other than body patterning. However, genetic evidence provides convincing support of the independent origins of these two species, with ∼7.2% mtDNA divergence and strongly supported differentiation for the nuclear gene RAG1.

The western Darling Downs species, from the Roma-area, was originally included in our study as the geographically closest population of *T. tetraporophora* sensu stricto to the Darling Downs earless dragon. The two specimens from the Roma-area (QMJ89119 & QMJ 87307) had been collected by S. Wilson during biodiversity surveys and additional tail tips and photographs in life had been taken. Thus, this important documentation, along with our genetic work, has uncovered an additional species in these grasslands. Our morphological examination of the specimens showed that they lacked femoral pores, thus, distinguishing them from *T. tetraporophora* sensu stricto ∼300 km further north-west at Tambo (Fig. 1). Based on this new information, the conservation status and management of this group in south-eastern Queensland needs to be revised.

Earless dragons are currently known from only a few sites within the Darling Downs region (Fig. 1) and are restricted to what were previously native grasslands. The Darling Downs is an important agricultural area on the western slopes of the Great Dividing Range in southern Queensland. Prior to European settlement, it was an area characterized by open prairie-like grasslands grading into brigalow (*Acacia harpophylla*)/belah (*Casuarina cristata*) forests [20]. The composition of the grasslands varies, with those in the east being dominated by bluegrass (*Dichanthium sericeum*) and those in the Roma-area (west) being Mitchell grass grasslands (*Astrebla* spp.). Most of the grasslands occur on cracking clay soils (vertosols), in particular black vertosols on alluvial plains and fans derived from basaltic parent material in the eastern Darling Downs [21]. An apparent exception to this are the sites west of Roma, which are also grasslands on dark colored vertosols, but parent material is Cretaceous mudstone (Doncaster Formation). These fertile soils have been heavily modified since European settlement and very little native grassland remains, making this one of the most threatened ecosystems in Queensland.

The disjunct distribution of the earless dragon species in the Darling Downs probably reflects the historical distributions of the grasslands, which were mostly absent from the central region. In fact, this historical disjunction of grasslands in the Darling Downs has probably shaped the divergence of these two species. There are some small areas of native grasslands along the Condamine River floodplain south of Chinchilla (C. Eddie, pers. comm.) and there are anecdotal reports of earless dragons from this area but no confirmed sightings (Hobson, pers. com.). Consequently, there is a need for detailed surveys of grasslands across the region to determine the full distribution of the two earless dragon species. In addition, further population genetics studies would help determine the connectivity of remnant habitats, inbreeding levels and whether these species have undergone recent bottlenecks. Although our study provides only the first, basic information about the Roma-area earless dragon, if it is anything like the eastern species - occurring in habitat remnants, croplands and roadside verges in highly fragmented populations - there is a strong probability that it will also be a species of significant conservation concern.

## Systematics

### *Tympanocryptis condaminensis* sp. nov. ZooBank LSID: urn:lsid:zoobank.org:act: 84833372-FA63-45B4-9B9D-FC9EB989FF65

Condamine Earless Dragon

(Figs. 7 & 8)

**Figure 7:**
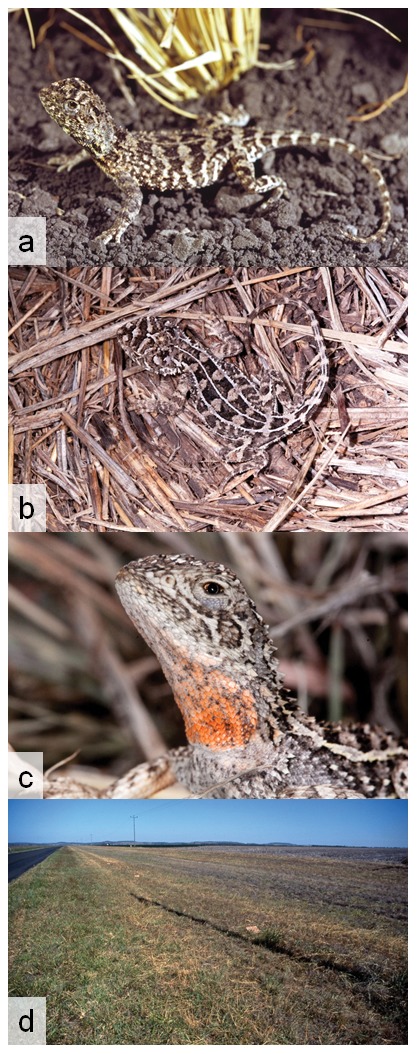
Photographs in life and habitat of *Tympanocryptis condaminensis* sp. nov. (a) Kunari Station, Darling Downs; (b) & (c) Bongeen, Darling Downs (photos S. Wilson); and (d) typical habitat - Bongeen, Darling Downs (photo S. Wilson).

**Figure 8:**
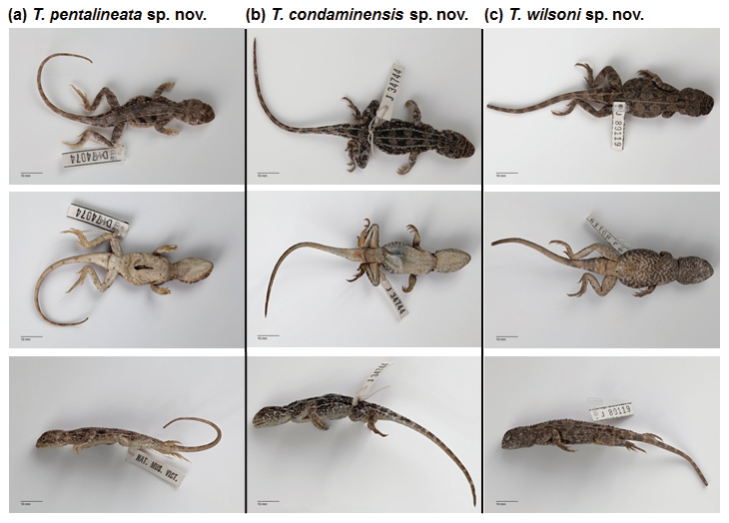
Holotypes of: (a) *Tympanocryptis pentalineata* sp. nov.; (b) *Tympanocryptis condaminensis* sp. nov.; and (c) *Tympanocryptis wilsoni* sp. nov. Dorsal, ventral and lateral views are shown for each holotype. Details on types series for these species are in Table 3.

#### Holotype

QMJ34744 *male* Oakey, 30 km South, Queensland, Australia (27° 39′ S, 151° 36′ E).

#### Paratypes

(4 specimens) QMJ81784 *male* Mt Tyson, Bongeen area, Queensland, Australia (27° 35′ S, 151° 33′ E); QMJ81870 *female* Kunari, via Bongeen, Queensland, Australia (27° 32′ 6″ S, 151° 26′ 46″ E); QMJ81871 *female* Kunari, via Bongeen, Queensland, Australia (27° 32′ 6″ S, 151° 26′ 46″ E); QMJ82087 *male* Brookstead, Queensland, Australia (27° 42′ 59″ S, 151° 28′ 20″ E).

#### Diagnosis

A small to medium-sized *Tympanocryptis* with a well-developed lateral and ventral body patterning, consisting of strongly contrasting brown-black and white irregular banding and speckling. Ventral patterning is concentrated on the head, throat and upper chest, extending posteriorly toward the lateral portions of the belly. Individuals with heavy ventral patterning usually have a narrow longitudinal stripe of white along the centreline of the upper third of the chest. Distinct irregular, dorso-ventral banding of brown-black and white colouration along the sides below a narrow but continuous white lateral stripe, which runs from axilla to groin. Ventral and lateral contrasting patterning consists of more white than brown-black. Three well defined pale spots on dorsal surface of snout: one above each nostril and one at end of snout. Inter-nasal scales >10. Scales on dorsal surface of the torso are heterogeneous with interspersed un-keeled, weakly keeled and strongly keeled scales. A distinct narrow white stripe running along the posterior edge of the thigh, extending onto the base of the tail. Femoral pores absent; preanal pores 2.

#### Description of holotype

A small dragon with a bluntly rounded snout, a gular fold, and a scapular fold. The scales on the dorsal surface of the head, body and tail are heterogeneous with interspersed un-keeled, weakly keeled and strongly keeled scales; scattered enlarged, mucronate scales. Ventral scales are un-keeled to weakly keeled on head and throat, weakly keeled on belly and strongly keeled on tail. A somewhat distinct neck, limbs slender and moderately long; canthus well defined; nasal scale below canthal ridge, nare slightly to the posterior-dorsal section of the nasal scale. Scales on dorsal surface of head keeled with three well defined pale spots on dorsal surface of snout: one above each nostril and one at end of snout. Scattered enlarged, keeled mucronate scales present on side of head posterior to the eye. Labials elongate and keeled. Tail long and slender, tapering distinctly approximately 1/3^rd^ along its length to a fine tip.

Supralabials 11, infralabials 10; inter-nasal 11; sub-ocular 10; scales between nasal and supralabial 4; no visible tympanum; two preanal pores; femoral pores absent.

In preservative, body patterning is strongly contrasting brown-black and white irregular banding and speckling. Black ventral speckling is concentrated on the head, throat and upper chest, extending posteriorly toward the lateral portions of the belly. A narrow longitudinal stripe of contrasting white is present along the centreline of the upper third of the chest. Distinct irregular, dorso-ventral banding of brown-black and white colouration along the sides below a narrow but continuous white lateral stripe, which runs from axilla to groin. Ventral and lateral contrasting patterning consists of more white than brown-black. Three well defined pale spots on dorsal surface of snout: one above each nostril and one at end of snout.

#### Variation

Tables 1 and 3 present variation in morphological and meristic characters within *T. condaminensis* **sp. nov.** No obvious differences were apparent in the patterning between males and females, except that males had slightly less ventral colouration.

**Table 3:** **Summary of meristic (mm) and mensural data for the types of** ***T. condaminensis*** **sp. nov.,** ***T. wilsoni*** **sp. nov.,** ***T. penatalineata*** **sp. nov., and** ***T. tetraporophora*** **.**

	*T. tetraporophora*	*T. condaminensis* sp. nov.					*T. wilsoni* sp. nov.		*T. pentalineata* sp. nov.		
	NMV7701 Lectotype	QMJ34744 Holotype	QMJ81784 Paratype	QMJ81870 Paratype	QMJ81871 Paratype	QMJ82087 Paratype	QMJ89119 Holotype	QMJ87307 Paratype	NMVD74073 Holotype	NMVD74074 Paratype	NMVD74075 Paratype
Sex	Male	Male	Male	Female	Female	Male	Male	Juvenile	Male	Male	Female
SVL	51.21	51.00	63.49	53.34	63.30	45.49	53.74	40.06	50.89	46.8	59.09
HL	17.35	15.5	20.93	17.13	18.55	16.41	19.88	12.68	17.11	16.81	18.62
HW	12.10	10.9	14.08	12.92	13.52	10.79	13.11	10.39	11.8	11.4	13.59
HD	9.18	7.95	9.07	8.51	9.59	7.53	9.35	6.06	7.95	7.4	8.63
SL	14	11	13	10	12	13	11	11	10	9	11
IL	16	10	12	11	12	11	12	12	12	10	12
IN	8	9	12	10	8	11	9	8	6	6	7
NS	6	4	5	4	5	5	4	4	5	4	5
SO	13	10	12	12	11	12	12	10	12	12	12
AG	27.54	22.6	33.74	23.77	31.72	27.03	30.66	24.56	28.61	24.92	32.8
LegL	42.65	39.35	46.52	37.83	37.39	34.27	40.32	28.88	34.94	35.23	41.79
SLam	19	18	17	18	19	19	19	18	15	17	17
TailL	76.98	79.6	98.01	71.65	73.02	65.89	63.14^a^	broken	82.04	74.47	95.72
PP	2	2	2	2	1	2	2	2	2	2	2
FP	2	0	0	0	0	0	0	0	2	2	2

a tip of tail missing.

SVL =  snout-vent length; HL =  head length; HW =  head width; HD =  head depth; SL =  supralabials; IL =  infralabials; IN =  inter-nasals; NS =  scales between nasal and supralabial; SO =  sub-ocular; AG =  axilla to groin length; LegL =  hind limb length; SLAM =  4^th^ toe sub-digital lamellae; TailL =  tail length; PP =  pre-anal pores; FP =  femoral pores.

#### Habitat

Occurs in the remnant native grasslands, croplands and roadside verges of the eastern Darling Downs. These grasslands occur on black cracking clays of the Condamine River floodplain.

#### Distribution

Currently known from the eastern Darling Downs, as far north as the Pirrinuan/Jimbour area, west as far as the town of Dalby and south to the township of Clifton. To the east it has been recorded to the eastern extremity of the Darling Downs in the Aubigny/Purrawunda area on the western outskirts of Toowoomba. Specific locations include: Oakey, Mt Tyson, Brookstead, Bongeen, and Bowenville.

#### Etymology

Named for the Condamine River and its floodplain on which this species occurs.

#### Remarks

**Figure 9:**
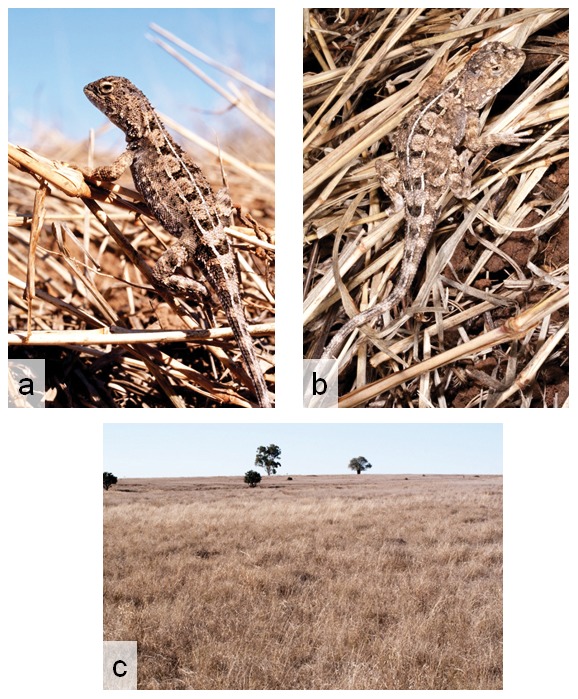
Photographs in life and habitat of *Tympanocryptis wilsoni* sp. nov., 40 km west Roma, Darling Downs. Photos by S. Wilson.

The distribution of *T. condaminensis* **sp. nov.** is not believed to overlap with any other *Tympanocrpytis*, however, its distribution is geographically close to *T. wilsoni* **sp. nov**, which occurs in the Roma area, west of the Darling Downs. It is believed that both these species are restricted to grasslands and do not occur in the habitats between the Darling Downs and Roma-area grasslands. Thus, they are not believed to be sympatric at any locations. There is an anecdotal record of a *Tympanocryptis* from a small isolated area of grassland in the Chinchilla/Condamine area, which is midway between Roma and the Darling Downs (Hobson, *pers. com.*). This isolated grassland provides an important avenue for future research into the distributions of these species. *T. condaminensis* **sp. nov.** can be distinguished from *T. wilsoni* **sp. nov** by the presence of a narrow white lateral stripe from axilla to groin and well-developed lateral and ventral body patterning, consisting of strongly contrasting brown-black and white irregular banding and speckling with more white that brown-black colouration. *T. wilsoni* **sp. nov** also has strong ventral and lateral patterning but it doesn't form irregular contrasting bands, there is more black-brown than white colouration, and the lateral stripe is absent. It is also known that some individuals of *T. condaminensis* **sp. nov.** have red-pink colouration on their throats (Fig. 6c), it is not currently known what this is related to (e.g., sexual colouration, breeding condition etc.). In addition, some individuals have been found to have lemon yellow along their sides, similar to that seen in some *T. tetraporophora* sensu stricto (Fig. 9a).

### *Tympanocryptis pentalineata* sp. nov. ZooBank LSID: urn:lsid:zoobank.org:act: 86F7CB90-DFAA-45DC-B755-9FCDF470A5AB

Five-lined earless dragon

(Figs. 8)

#### Holotype

NMVD74074 *male* 47.9 km south-west of the intersection between the Burke Development Rd (Route 83) and the Gulf Development Rd (Route 1), Queensland (18° 06′ 29″ S, 140° 53′ 00″ E).

#### Paratypes

(2 specimens) NMVD74073 *male*, 47.9 km south-west of the intersection between the Burke Development Rd (Route 83) and the Gulf Development Rd (Route 1), Queensland (18° 06′ 29″ S, 140° 53′ 00″ E); NMVD74075 *female*, 47.9 km south-west of the intersection between the Burke Development Rd (Route 83) and the Gulf Development Rd (Route 1), Queensland (18° 06′ 29″ S, 140° 53′ 00″ E).

#### Note on type locality

Original written description of the locality states “Gulf Development Road, 50 km south of Normanby”, examination of the GPS record of 18° 06′ 29″ S, 140° 53′ 00″ E indicates that the location is actually on the Burke Development Rd, 47.9 km south-west of the intersection between the Burke Development Rd (Route 83) and the Gulf Development Rd (Route 1).

#### Diagnosis

A medium-sized *Tympanocryptis* with a distinct dorsal body pattern, consisting of five longitudinal narrow grey or white stripes on a brown-black patterned background. The five stripes consist of: one weak, narrow, grey vertebral stripe; two white, narrow dorso-lateral stripes; and two narrow, white lateral stripes. When examined closely the lateral stripes consist of a single row of enlarged, mucronate white scales extending from axilla to groin, bordered ventrally and dorsally with smaller, darker scales. Strong background patterning is evident between the dorso-lateral and lateral stripes, consisting of three broad transverse bands that are dark-brow to black. Scattered through the darker background colouring are enlarged white mucronate scales, giving the impression of white flecks on a dark background. The dorso-lateral stripes continue onto the tail to about 1/3^rd^ of its length. There is a cluster of 3–5 enlarged, pale mucronate scales at the anterior extent of the paravertebral stripes, sitting at the rear of the head. On the ventral surface, the throat and upper chest area is faintly pigmented with black flecks or pigmentation is absent. Nare slightly off-centre towards the posterior-dorsal section of the nasal scale. Four pores, two pre-anal pores and two femoral pores.

#### Description of holotype

A slender dragon with a bluntly rounded snout, a gular fold, and a scapular fold. The scales on the dorsal surface of the head, body and tail are strongly keeled and the scales on the ventral surface are weakly-strongly keeled. A distinct neck, limbs slender and moderately long; canthus well defined; nasal scale below canthal ridge, nare slightly to the posterior-dorsal section of the nasal scale. Tail long and slender, tapering distinctly approximately 1/3^rd^ along its length to a fine tip.

Supralabials 9, infralabials 10; inter-nasal 7; sub-ocular 12; scales between nasal and supralabial 4; no visible tympanum. Labials elongate and strongly keeled. Scales on dorsal surface of head homogeneous and strongly keeled but without distinct pale band between eyes or nasal scales. Scattered enlarged, keeled mucronate scales present on side of head posterior to the eye. Dorsals heterogeneous, strongly keeled, diamond-shaped with scattered enlarged, mucronate scales; ventral scales homogeneous and strongly keeled; 2 preanal pores; and two femoral pores, one on each side.

Body pattern consists of five longitudinal narrow grey-white stripes on a brown-black patterned background, consisting of one weak, narrow, grey vertebral stripe; two white, narrow dorso-lateral stripes; and two narrow, white lateral stripes. Lateral stripe consists of a single row of enlarged, mucronate white scales extending from axilla to groin, bordered ventrally and dorsally with smaller, darker scales. Strong background patterning is evident between the dorso-lateral and lateral stripes, consisting of three broad transverse bands that are dark-brow to black. Scattered throughout dark colouring on dorsum are enlarged white mucronate scales, giving the impression of white flecking. The dorso-lateral stripe continues onto the tail to about 1/3^rd^ of its length.

#### Variation

Tables 1 and 3 present variation in morphological and meristic characters within *T. pentalineata* **sp. nov.** No obvious differences were apparent in the patterning between males and females, except that males had slightly more ventral colouration, with more black flecking on the throat and upper chest.

#### Habitat

Occurs on flat flood-plains, covered by grasses and low perennial shrubs.

#### Distribution

Currently only known from the one location, 50 km south-west of Normanton in the gulf region of far northern Queensland (Fig. 1).

#### Etymology

Named for the dorsal colour pattern of the new species, characterised by five longitudinal white stripes extending along the body, one vertebral, two dorso-lateral and two lateral.

#### Remarks

The distribution of *T. pentalineata* **sp. nov.** potentially overlaps with a number of other *Tympanocryptis*, including *T. tetraporophora* Lucas and Frost 1895 (specimens having been collected from approximately 200 km away at 17° 56′ 06″ S, 139° 18′ 07″ E), *T. lineata* (with the junior synonym *T. karumba* Wells and Wellington 1985 from Karumba, QLD (17°29′ S, 140°50′ E)), *T. cf lineata* collected on the road to Esmeralda, 50 km S of Gulf Development Road (18° 33′ 43″ S, 142° 34′ 04″ E) and *T. cf intima* collected from the Gulf Development Road, 130 km S of Normanby (18° 40′ 37″ S, 140° 30′ 42″ E). DNA sequencing has confirmed that the *T. tetraporophora, T. cf lineata* (Melville, unpub. data) and *T. cf intima* (Melville, unpub. data) from the region are unrelated to *T. pentalineata* **sp. nov.** Additionally, *T. cf lineata* and *T. cf intima* collected from the region occurred in different habitats to *T. pentalineata* **sp. nov.**: *T. cf lineata* specimens were collected from low rock outcrops, while *T. cf intima* were collected from flat stony ground with scattered spinifex. Morphologically, *T. pentalineata* **sp. nov.** can be distinguished from these species, with the combination of two femoral and two preanal pores, five longitudinal stripes, and dorsal scales heterogeneous, strongly keeled, diamond-shaped with scattered enlarged, mucronate scales.

### *Tympanocryptis wilsoni* sp. nov. ZooBank LSID: urn:lsid:zoobank.org:act: 15F26C63-5208-4DEA-AFA3-390BA935707B

Roma earless dragon

(Figs. 8 & 9)

#### Holotype

QMJ89119 *male* Mount Abundance Rd, 40 km south-west of Roma, Queensland, Australia (26° 42′ 10″ S, 148° 29′ 10″ E).

#### Paratype

(1 specimen) QMJ87307 *juvenile* Cherax Flats, Hodgson, Queensland, Australia (26° 34′ 25″ S, 148° 38′ 38″ E).

#### Note on type locality

Original written description of the locality states “Mount Abundance Rd, 40 km East of Roma”, Mount Abundance Rd is west of Roma and examination of the GPS record of 26° 42′ 10″ S, 148° 29′ 10″ E indicates that the location is 40 km south-west of Roma.

#### Diagnosis

A small to medium-sized *Tympanocryptis* with a well-developed lateral and ventral body patterning, consisting of extensive brown-black speckling. Ventral patterning is concentrated on the head, throat and upper chest, extending posteriorly toward the lateral portions of the belly. Heavy brown-black speckling along the sides but white lateral stripe is absent. Ventral and lateral patterning black-brown colouration is greater than white. Three well defined pale spots on dorsal surface of snout: one above each nostril and one at end of snout. Inter-nasal scales <10. Scales on dorsal surface of the torso are heterogeneous with interspersed un-keeled, weakly keeled and strongly keeled scales. Femoral pores absent; preanal pores 2.

#### Description of holotype

A small dragon with a bluntly rounded snout, a gular fold, and a scapular fold. The scales on the dorsal surface of the head, body and tail are heterogeneous with interspersed un-keeled, weakly keeled and strongly keeled scales; scattered enlarged, mucronate scales. Ventral scales are un-keeled to weakly keeled on head and throat, weakly keeled on belly and strongly keeled on tail. A somewhat distinct neck, limbs slender and moderately long; canthus well defined; nasal scale below canthal ridge, nare slightly to the posterior-dorsal section of the nasal scale. Scales on dorsal surface of head keeled with three well defined pale spots on dorsal surface of snout: one above each nostril and one at end of snout. Scattered enlarged, keeled mucronate scales present on side of head posterior to the eye. Labials elongate and keeled. Tail long and slender, tapering distinctly approximately 1/3^rd^ along its length to a fine tip.

Supralabials 11, infralabials 12; inter-nasal 9; sub-ocular 12; scales between nasal and supralabial 4; no visible tympanum; two preanal pores; femoral pores absent.

Dark brown-black lateral and ventral body speckling. Ventral patterning is concentrated on the head, throat and upper chest, extending posteriorly toward the lateral portions of the belly. Heavy brown-black speckling along the sides but white lateral stripe is absent. Ventral and lateral patterning black-brown colouration is proportionally greater than white background. Three well defined pale spots on dorsal surface of snout: one above each nostril and one at end of snout.

#### Variation

Tables 1 and 3 present variation in morphological and meristic characters within *T. wilsoni* **sp. nov.** No obvious differences were apparent in the patterning between the two specimens.

#### Habitat

Currently known to occur in grasslands on sloping terrains. Grasslands in the western Darling Downs are dominated by Mitchell grasses.

#### Distribution

Based on four known samples (2 x vouchers + tissues & 2 x tissues only), the distribution of this species is in the native grasslands nearby the town of Roma: from Hodgson approximately 20 km west to Mt Abundance approximately 50 km south-west of Roma.

#### Etymology

Named in recognition of the contributions of Steve Wilson to Australian herpetology, in particular his direct contribution to the understanding of *Tympanocryptis* diversity in Queensland. Steve Wilson discovered this new species during a survey, provided photographs in-life and collected the only voucher specimens.

#### Remarks

Based on only four specimens, little is known of the distribution of *T. wilsoni* **sp. nov.**, however, its distribution is geographically close *T. condaminensis* **sp. nov.** which occurs further east in the Darling Downs. To the west and south of the known distribution of *T. wilsoni* **sp. nov.** occurs *T. tetraporophora* sensu stricto, including specimens collected from near St George (28° 28′ 3″ S, 148° 43′ 8″ E) approximately 200 km south of Roma and from 30 km south of Tambo (25° 7′ 34″ S, 146° 6′ 18″ E), which is approximately 300 km north-west of Roma. Molecular work confirmed that the specimens from around Tambo (QMJ84184-QMJ84187) are *T. tetraporophora* sensu stricto and an independent genetic lineage in both mtDNA and nuclear DNA (RAG1) from *T. wilsoni* **sp. nov.**. In addition, *T. wilsoni* **sp. nov.** can be easily distinguished from nearby *T. tetraporophora* sensu stricto by the absence of femoral pores.

### Tympanocryptis tetraporophora

Eyrean earless dragon

Lucas and Frost 1895

(Figs 10 & 11)

**Figure 10:**
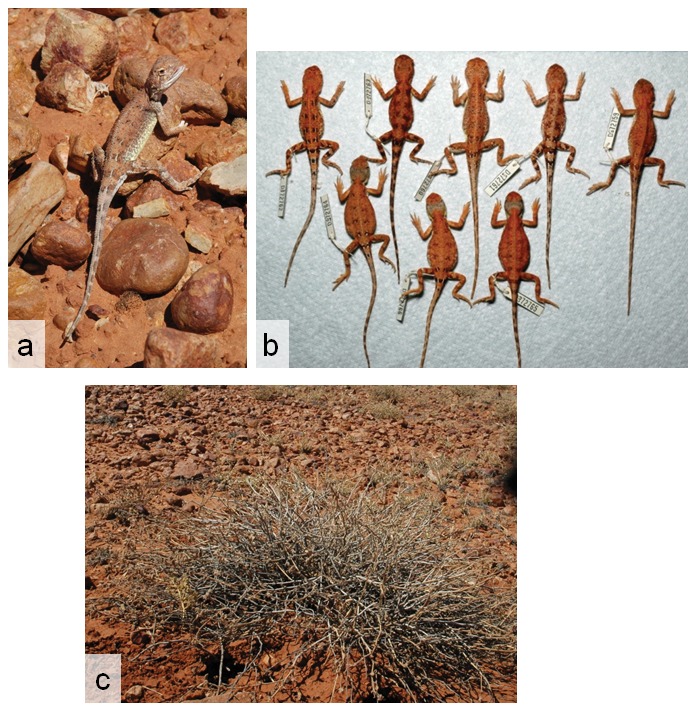
Photographs in life, specimens before preservation (showing colour and pattern variation) and habitat of *Tympanocryptis tetraporophora* sensu stricto from Mt Dare, Northern Territory, which is in the topotypic region. MtDNA phylogeny indicates these individuals are in the same clade as the Lectotype NMVD7701 (Fig. 2a). In image (b) top row are males (x 5) and bottom row are females (x 3). Photos by R. Glor.

**Figure 11:**
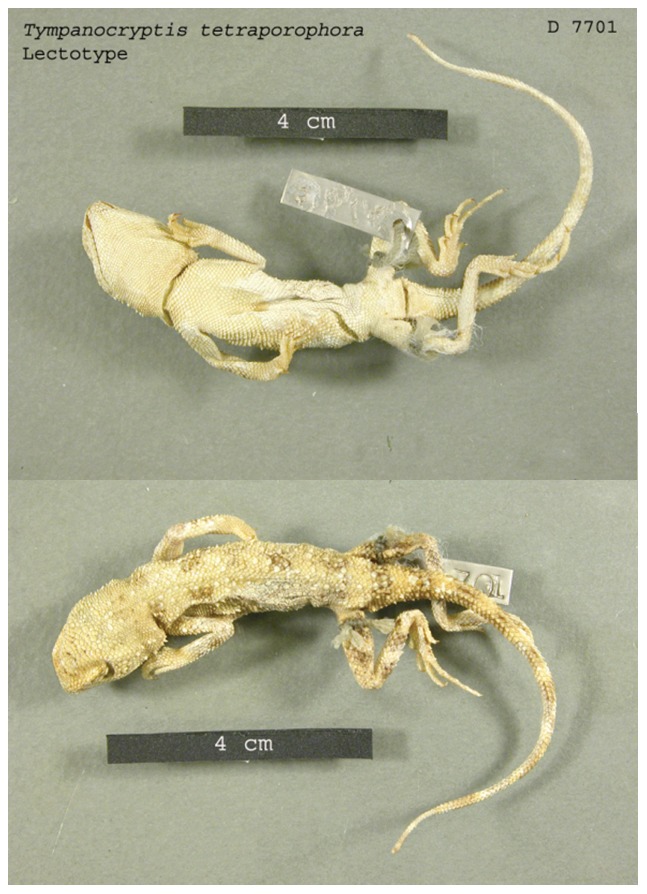
Lectotype of *Tympanocryptis tetraporophora* (NMVD7701): dorsal and ventral view.

#### Lectotype

NMVD7701 *male* Adminga and Dalhousie, SA. Collected on the Horn Expedition into central Australia in 1894.

#### Diagnosis

A medium-sized *Tympanocryptis* with very variable body patterning, consisting of darker brown broken cross-bands on body. A weak to strong single pale ventral stripe. Dorsal scales homogenous with scattered enlarged mucronate scales, enlarged scales not substantially larger than other scales. Dorsal head scales homogenous, weakly-strongly keeled with scattered enlarged mucronate scales mainly to rear of head and neck. Tail scales strongly keeled and tail tapers to rounded tip. Dorsal patterning variable, 4–6 broken darker cross-bands on torso on a reddy-brown or brown background. Dark brown bands on legs and also extending down 2/3^rds^ of the tail length. Banding does not extend to ventral surfaces. Ventral scales weakly to strongly keeled. Some individuals have dark pigmentation on ventral surface of head, throat and upper chest. Nostril nearer eye than tip of snout. Four pores present, two pre-anal and two femoral pores.

#### Description of holotype

A slender dragon with a bluntly rounded snout, a gular fold, and a scapular fold. The scales on the dorsal surface of the head, body and tail are strongly keeled and the scales on the ventral surface are weakly-strongly keeled. A distinct neck, limbs slender and moderately long; canthus well defined; nasal scale below canthal ridge and closer to eye than tip of snout, nare slightly to the posterior-dorsal section of the nasal scale. Tail tapering distinctly approximately 1/3^rd^ along its length to a rounded tip.

Supralabials 14, infralabials 16; inter-nasal 8; sub-ocular 13; scales between nasal and supralabial 6; no visible tympanum. Labials elongate and weakly keeled. Scales on dorsal surface of head homogeneous and strongly keeled but without distinct pale band between eyes or nasal scales. Scattered enlarged, keeled mucronate scales present on side of head posterior to the eye. Dorsals heterogeneous, strongly keeled, scattered enlarged, mucronate scales; enlarged scale are not substantially larger than other scales. Ventral scales homogeneous and strongly keeled on body and tail, weakly keeled under head; two preanal pores; and two femoral pores, one on each side.

Little body patterning apparent in preservation, consists of four dark broken cross-bands on body between the limbs. Dark banding on the hind legs, two on thighs and two on lower legs; banding does not extend to ventral surface. Six bands present on tail, which do not extend to ventral surface.

#### Variation

Tables 1 and 3 present variation in morphological and meristic characters within *T. tetraporophora*. Measurements of lectotype in current study closely reflect those in original species description. Body patterning very variable and clear differenced can exist between males and females, with some females having very little body patterning and a distinct grey band at the back of the head (see Fig. 9).

#### Habitat

Broad range of habitats, from stony desert plains, inland floodplains, black soil plains to tropical savannah grasslands.

#### Distribution

A very large distribution across a wide climatic gradient, ranging from the arid interior of South Australia, to semi-arid New South Wales and into the tropical grasslands of the Gulf region in northern Queensland.

#### Etymology

Named for the presence of four pores, two pre-anal and two femoral.

#### Remarks

The distribution of *T. tetraporophora* overlaps with a number of other *Tympanocryptis*, including *T. cephalus*, *T. lineata, T. intima* and *T. pentalineata* **sp. nov.** *T. tetraporophora* can be distinguished from the former three species by the presence of four pores (two preanal and two femoral). *T. tetraporophora* can be distinguished from *T. pentalineata* **sp. nov** by lacking five longitudinal body stripes.

## Supporting Information

Table S1 Locality information and GENBANK accession numbers for all individuals sampled in this study.
(PDF)

Appendix S1 List of specimens examined morphologically.
(PDF)

## References

[1] Knowlton N (1993) Sibling species in the sea. Annu Rev Ecol Evol Syst 24: 189–216.

[2] Kozak KH, Weisrock DW, Larson A (2006) Rapid lineage accumulation in a non-adaptive radiation: phylogenetic analysis of diversification rates in eastern North American woodland salamanders (Plethodontidae: Plethodon). Proc R Soc Lond B 273: 539–546. 10.1098/rspb.2005.3326 PMC1560065 16537124

[3] Miller SE (2007) DNA barcoding and the renaissance of taxonomy. Proc Nat Acad Sci USA 104: 4775–4776. 17363473 10.1073/pnas.0700466104 PMC1829212

[4] Wiens JJ, Penkrot TA (2002) Delimiting species using DNA and morphological variation and discordant species limits in spiny lizards (*Sceloporus*). Syst Biol 51: 69–91. 11943093 10.1080/106351502753475880

[5] Miralles A, Vences M (2013) New metrics for comparison of taxonomies reveal striking discrepancies among species delimitation methods in *madascincus* lizards. PLoS ONE 8(7): e68242 doi:10.1371/journal.pone.0068242 23874561 10.1371/journal.pone.0068242 PMC3710018

[6] Greer AE (1989) The biology and evolution of Australian lizards. Surrey Beatty and Sons, Sydney, New South Wales, Australia.

[7] Mitchell F (1948) A revision of the Lacertilian genus *Tympanocryptis* . Rec South Aust Mus 9: 57–86.

[8] Melville JE, Keogh S, Starr C, Austin J (2007) Phylogeography, conservation and species status of an endangered Australian dragon, *Tympanocryptis pinguicolla* (Reptilia: Agamidae). Conserv Genet 8: 185–195.

[9] Smith WJ, Osborne WS, Donnellan SC, Cooper PD (1999) The systematic status of earless dragon lizards, *Tympanocryptis* (Reptilia: Agamidae) in south-eastern Australia. Aust J Zool 47: 551–564.

[10] Starr CR, Leung LK-P (2006) Habitat use by the Darling Downs population of the grassland earless dragon: implications for conservation. J Wild Man 70: 897–903.

[11] Austin JJ, Melville J (2006) Incorporating historical museum specimens into molecular systematic and conservation genetics research. Mol Ecol Notes 6: 1089.

[12] Melville J, Hale J (2009) Length variation in the N-terminal domain of the recombination-activating gene 1 (RAG1) across squamates. Mol Phyl Evol 52: 898–903. 10.1016/j.ympev.2008.12.027 19603552

[13] Melville JE, Richie G, Chapple SNJ, Glor RE, Schulte II JA (2011) Evolutionary origins and diversification of dragon lizards in Australia's tropical savannas. Mol Phyl Evol 58: 257–270. 10.1016/j.ympev.2010.11.025 21145401

[14] Ronquist F, Huelsenbeck JP (2003) MRBAYES 3: Bayesian phylogenetic inference under mixed models. Bioinformatics 19: 1572–1574. 12912839 10.1093/bioinformatics/btg180

[15] Tavaré S (1986) Some probabilistic and statistical problems in the analysis of DNA sequences. Lect Math Life Sci 17: 57–86.

[16] Nylander JAA (2004) MrModeltest v2. Program distributed by the author. Evolutionary Biology Centre, Uppsala University, Sweden.

[17] Rambaut A, Drummond AJ (2007) Tracer v1.4, Available: http://tree.bio.ed.ac.uk/software/tracer. Accessed 2014 Jul 3.

[18] Heled J, Drumond AJ (2010) Bayesian inference of species trees from multilocus data. Mol Biol Evol 27: 570–580. 19906793 10.1093/molbev/msp274 PMC2822290

[19] Covacevich JA, Couper PJ, McDonald KR (1998) Reptile diversity at risk in the Brigalow Belt, Queensland. Memoirs Qld Mus 42: 475–486.

[20] Silcock RG, Scattini WJ (2007) The original native pasture ecosystems of the eastern and western Darling Downs — can they be restored? Tropical Grasslands 41: 154–163.

[21] Harris PS, Biggs AJW, Stone BJ (1999) Central Darling Downs Land Management Manual. Department of Natural Resources, Queensland, Australia.

[22] Wilson S, Swan G (2013) A Complete Guide to Reptiles of Australia, 4^th^ ed. Reed New Holland, Sydney, Australia.

