# Supplementary material for: The Role of Integrative Taxonomy in the Conservation Management of Cryptic Species: The Taxonomic Status of Endangered Earless Dragons (Agamidae: Tympanocryptis) in the Grasslands of Queensland, Australia
Source: PLoS One. 2014 Jul 30;9(7):e101847. doi: 10.1371/journal.pone.0101847 (PMC4116116; doi:10.1371/journal.pone.0101847)
Supplement: Table S1 — Locality information and GENBANK accession numbers for all individuals sampled in this study. (PDF) [file pone.0101847.s001.pdf]

Table S1. Museum registration numbers, localities of specimens from which DNA was extracted and GENBANK accession numbers are provided below. Museum acronyms are: AM for Australian Museum Sydney, QM for Queensland Museum, NMV for Museum Victoria Melbourne, SAMA for South Australian Museum, NTM for Northern Territory Museum and Art Gallery, ANWC for Australian National Wildlife Collection.

Museum ID	Species	Location	GPS	ND2 #	RAG1 #
QMJ81784	T. condaminensis	Mt Tyson, Bonjeen Area, QLD	27 35' S 151 33 E	KJ881036	KJ881150
QMJ81870	T. condaminensis	Kunari, Via Bongeen, QLD	27 32' 6' S 151 26' 46' E	KJ881037	KJ881151
QMJ82087	T. condaminensis	Brookstead, QLD	27 42' 59' S 151 28' 20' E	KJ881038	KJ881153
QMJ82088	T. condaminensis	Brookstead, QLD	27 42' 59' S 151 28' 20' E	KJ881039	KJ881154
QMJ81871	T. condaminensis	Kunari, Via Bongeen, QLD	27 32' 6' S 151 26' 46' E	KJ881040	KJ881152
NMVD74073	T. pentalineata	Gulf Development Rd, 50 Km South Of Normanby, QLD	18.11 S 140.88 E	KJ881041	KJ881143
NMVD74075	T. pentalineata	Gulf Development Rd, 50 Km South Of Normanby, QLD	18.11 S 140.88 E	KJ881042	
NMVD74074	T. pentalineata	Gulf Development Rd, 50 Km South Of Normanby, QLD	18.11 S 140.88 E	KJ881043	KJ881144
QM-A007430	T. wilsoni	Studley, QLD	26 42' 3.528' S 148 28' 19.812' E	KJ881032	KJ881155
QMJ87307	T. wilsoni	Cherax Flats, Hodgson, QLD	26 34' 25' S 148 38' 38' E	KJ881033	KJ881157
QM-A007431	T. wilsoni	Studley, QLD	26 42' 3.528' S 148 28' 19.812' E	KJ881034	KJ881156
QMJ89119	T. wilsoni	Mount Abundance Rd, 40 Km E Roma	26 42' 10' S 148 29' 10' E	KJ881035	KJ881158
NMVD7701 (Lectotype)	T. tetraporophora	Adminga Or Dalhouse, SA	26.12 S 134.85 E	KJ881067	
SAMAR45265	T. tetraporophora	20k NW Tilpa, NSW	31.2 S 144.55 E	KJ881021	
QMJ63876	T. tetraporophora	Isis Downs, Via Isisford, QLD	24 24' S 144 33' E	KJ881022	KJ881147
QMJ83530	T. tetraporophora	Australia:Whitehill Stn, QLD	21 6' 59' S 142 44' 44' E	KJ881023	
QMJ82191	T. tetraporophora	Diamantina Np, QLD	23 46' 47' S 141 11' 40' E	KJ881024	
QMJ74792	T. tetraporophora	Ernest Henry Mine Site, 20km N Cloncurry, QLD	20 26' 17' S 140 42' 45' E	KJ881025	KJ881145
QMJ74793	T. tetraporophora	Ernest Henry Mine Site, 20km N Cloncurry, QLD	20 26' 17' S 140 42' 45' E	KJ881026	KJ881146
QMJ83852	T. tetraporophora	Woolston, SE Richmond, QLD	21 6' 59' S 142 44' 44' E	KJ881027	
QMJ83854	T. tetraporophora	Woolston, SE Richmond, QLD	21 6' 59' S 142 44' 44' E	KJ881028	
QMJ84185	T. tetraporophora	Stirling Downs, 30 Km SSW Tambo, QLD	25 5' 41' S 146 6' 2' E	KJ881029	
QMJ84186	T. tetraporophora	Stirling Downs, 30 Km SSW Tambo, QLD	25 5' 29' S 146 4' 54' E	KJ881030	KJ881148
QMJ84187	T. tetraporophora	Stirling Downs, 30 Km SSW Tambo, QLD	25 5' 29' S 146 4' 54' E	KJ881031	KJ881149

NMVD72741	T. tetraporophora	20km From Hamilton Stn Hmstd, SA	26.66 S 135.22 E	KJ881044	KJ881122
NMVD72736	T. tetraporophora	20km From Hamilton Stn Hmstd, SA	26.66 S 135.22 E	KJ881045	
NMVD72753	T. tetraporophora	Near Dalhousie Springs, SA	26.66 S 135.22 E	KJ881046	KJ881127
NMVD72744	T. tetraporophora	17km From Hamilton Stn Hmstd, SA	26 39' 28.6' S 135 12' 13.3' E	KJ881047	
NMVD72752	T. tetraporophora	Mt. Dare, NT	25.98 S 135.09 E	KJ881048	
NMVD72755	T. tetraporophora	Mt. Dare, NT	25.98 S 135.09 E	KJ881049	
NMVD72756	T. tetraporophora	Mt. Dare, NT	25.98 S 135.09 E	KJ881050	
NMVD72757	T. tetraporophora	Mt. Dare, NT	25.98 S 135.09 E	KJ881051	
NMVD72758	T. tetraporophora	Mt. Dare, NT	25.98 S 135.09 E	KJ881052	
NMVD72760	T. tetraporophora	Mt. Dare, NT	25.98 S 135.09 E	KJ881053	
NMVD72761	T. tetraporophora	Mt. Dare, NT	25.98 S 135.09 E	KJ881054	
NMVD72762	T. tetraporophora	Mt. Dare, NT	25.98 S 135.09 E	KJ881055	
NMVD72763	T. tetraporophora	Mt. Dare, NT	25.98 S 135.09 E	KJ881056	
NMVD72764	T. tetraporophora	Mt. Dare, NT	25.98 S 135.09 E	KJ881057	
NMVD72765	T. tetraporophora	Mt. Dare, NT	25.98 S 135.09 E	KJ881058	
NMVD72766	T. tetraporophora	Mt. Dare, NT	25.98 S 135.09 E	KJ881059	
NMVD72770	T. tetraporophora	Mt Dare Stn, NT	25.98 S 135.10 E	KJ881060	KJ881123
NMVD72754	T. tetraporophora	Australia:Mt Dare Stn, NT	25.98 S 135.10 E	KJ881061	
SAMAR46999	T. tetraporophora	5k S Mosquito Camp Dam, SA	26.10 S 134.48 E	KJ881062	KJ881124
NMVD72759	T. tetraporophora	Mt. Dare, NT	25.98 S 135.09 E	KJ881063	
NMVD72768	T. tetraporophora	Near Dalhousie Springs, SA	25.98 S 135.10 E	KJ881064	
SAMAR38091	T. tetraporophora	5kms SE Mt. Crispe, SA	26.40 S 135.38 E	KJ881065	KJ881128
ANWCR6135	T. tetraporophora	54km East Muga Park Station, Mulga Pk Rd, NT	25.98 S 131.58 E	KJ881066	
SAMAR48423	T. tetraporophora	Allandale Stn, SA	27.62 S 135.58 E	KJ881068	
SAMAR44722	T. tetraporophora	Todmorden Stn, SA	27.13 S 134.75 E	KJ881069	
SAMAR26543	T. tetraporophora	Granite Downs H/S, SA	26.93 S 133.48 E	KJ881070	
SAMAR48505	T. tetraporophora	Nilpinna Stn, SA	28.20 S 135.68 E	KJ881071	
SAMAR47309	T. tetraporophora	Peake Stn, SA	28.23 S 135.90 E	KJ881072	KJ881125
SAMAR46481	T. tetraporophora	13.6k S Mungutana Dam, SA	29.33 S 135.68 E	KJ881073	

SAMAR26805	T. tetraporophora	25km S Mabel Ck H/S, SA	28.94 S 134.32 E	KJ881074	
SAMAR48380	T. tetraporophora	Mount Barry Stn, SA	28.23 S 134.98 E	KJ881075	KJ881126
SAMAR46538	T. tetraporophora	5km W Mt Margaret, SA	28.48 S 136.07 E	KJ881076	
SAMAR20871	T. tetraporophora	48k S Olympic Dam, SA	30.45 S 136.90 E	KJ881077	KJ881129
NMVD71386	T. tetraporophora	Trk To Corona Stn, NTH Of Broken Hill, NSW	31.44 S 141.49 E	KJ881078	KJ881130
NMVD71387	T. tetraporophora	Trk To Corona Stn, NTH Of Broken Hill, NSW	31.44 S 141.48 E	KJ881079	
NMVD71388	T. tetraporophora	Trk To Corona Stn, NTH Of Broken Hill, NSW	31.68 S 141.57 E	KJ881080	
NMVD71389	T. tetraporophora	Trk To Corona Stn, NTH Of Broken Hill, NSW	31.68 S 141.57 E	KJ881081	
NMVD71390	T. tetraporophora	Trk To Corona Stn, NTH Of Broken Hill, NSW	31.68 S 141.57 E	KJ881082	KJ881131
SAMAR41301	T. tetraporophora	Alderman's Catch, SA	32.53 S 140.62 E	KJ881083	KJ881132
SAMAR41504	T. tetraporophora	Manunda Ck, SA	32.77 S 139.65 E	KJ881084	KJ881133
SAMAR53153	T. tetraporophora	5k Nf Callory Bore, SA	31.80 S 138.75 E	KJ881085	
SAMAR51945	T. tetraporophora	1.5 Km Wsw Of Reedy Hole Springs, SA	30.25 S 138.83 E	KJ881086	
SAMAR46371	T. tetraporophora	2.5k W Wangianna Rail Siding, SA	29.65 S 137.70 E	KJ881087	KJ881134
AMR151151	T. tetraporophora	Sturt National Park, NSW	29.48 S 142.23 E	KJ881088	
AMR151146	T. tetraporophora	Sturt National Park, Binerah Downs, NSW	29.02 S 141.55 E	KJ881089	
AMR151144	T. tetraporophora	Sturt National Park, Binerah Downs, NSW	29.02 S 141.55 E	KJ881090	KJ881135
AMR153258	T. tetraporophora	Milparinka, NSW	29.72 S 141.87 E	KJ881091	
AMR151128	T. tetraporophora	Sturt National Park, 12 Mile Creek Crossing, NSW	29.20 S 141.97 E	KJ881092	
AMR151141	T. tetraporophora	Sturt National Park, NSW	29.05 S 141.85 E	KJ881093	
AMR152948	T. tetraporophora	Sturt National Park, NSW	29.05 S 141.85 E	KJ881094	
AMR151676	T. tetraporophora	White Cliffs, 34.7km From Silver City Hwy, NSW	30.83 S 143.08 E	KJ881095	
QMJ48542	T. tetraporophora	100km S Jackson, QLD	26.63 S 149.63 E	KJ881096	
SAMAR31167	T. tetraporophora	Innamincka Ruins, SA	27.73 S 140.73 E	KJ881097	KJ881136
SAMAR32468	T. tetraporophora	Innamincka, SA	27.73 S 140.73 E	KJ881098	
SAMAR46041	T. tetraporophora	Approx 30k SE Moomba Gas Field, SA	28.15 S 140.17 E	KJ881099	
SAMAR42934	T. tetraporophora	Eromanga Dump, QLD	26.33 S 142.87 E	KJ881100	KJ881137
AMR143897	T. tetraporophora	22.3km N Of Barkly Hwy Via Normonton Road, QLD	20.58 S 140.43 E	KJ881101	
AMR143850	T. tetraporophora	28km E Ilfracombe On Landsborough Hwy, QLD	23.60 S 144.58 E	KJ881102	

AMR143863	T. tetraporophora	0.7km Ne Winton, QLD	22.37 S 143.03 E	KJ881103	
SAMAR42734	T. tetraporophora	SE Of Muttaburra, QLD	22.58 S 144.52 E	KJ881104	KJ881142
SAMAR42806	T. tetraporophora	10 Km NW Of Diamantina Stn, QLD	23.77 S 141.13 E	KJ881105	
QMJ81203	T. tetraporophora	Myuna Stn Via Collinsville, QLD	21.07 S 143.22 E	KJ881106	
NMVD74051	T. tetraporophora	Road To Gregory Downs, 3km S Of	17.89 S 138.34 E	KJ881107	
NMVD74046	T. tetraporophora	8km S Of Carpentaria Highway, QLD	17.94 S 139.30 E	KJ881108	KJ881141
NMVD74049	T. tetraporophora	16km S Of Carpentaria Highway, QLD	17.87 S 139.34 E	KJ881109	
NMVD74048	T. tetraporophora	16km S Of Carpentaria Highway, QLD	17.87 S 139.34 E	KJ881110	
AMR147233	T. tetraporophora	52 Km N Barkly Roadhouse On Cape Crawford Rd, NT	19.70 S 135.82 E	KJ881111	
AMR147225	T. tetraporophora	104.8 Km W Camooweal (= 3.6 Km E Soudan), QLD	19.62 S 138.52 E	KJ881112	KJ881138
AMR147226	T. tetraporophora	104.8 Km W Camooweal (= 3.6 Km E Soudan), QLD	19.62 S 138.52 E	KJ881113	
AMR147236	T. tetraporophora	6 Km Along Old Barkly Stock Route, NT	17.98 S 135.18 E	KJ881114	KJ881139
NMVD72694	T. tetraporophora	Brunette Downs Hmstd Rd, Tablelands Hwy, NT	18.64 S 135.96 E	KJ881115	KJ881140
NMVD72739	T. tetraporophora	Brunette Downs Hmstd Rd, Tablelands Hwy, NT	18.64 S 135.96 E	KJ881116	
NTMR24487	T. tetraporophora	Nr Lake Corella Barkly Tableland, NT	18.72 S 135.59 E	KJ881117	
SAMAR54456	T. tetraporophora	Toorak Hs, QLD	21.03 S 141.78 E	KJ881118	
SAMAR55728	T. tetraporophora	7.5k E Julia Ck On Flinders Highway, QLD	20.65 S 141.81 E	KJ881119	
SAMAR54036	T. tetraporophora	Playford River Crossing, On Tablelands H/Way, NT	19.29 S 136.06 E	KJ881120	
SAMAR54453	T. tetraporophora	15k S Julia Creek, QLD	20.82 S 141.74 E	KJ881121	
