# Supplementary material for: The Role of Integrative Taxonomy in the Conservation Management of Cryptic Species: The Taxonomic Status of Endangered Earless Dragons (Agamidae: Tympanocryptis) in the Grasslands of Queensland, Australia
Source: PLoS One. 2014 Jul 30;9(7):e101847. doi: 10.1371/journal.pone.0101847 (PMC4116116; doi:10.1371/journal.pone.0101847)
Supplement: Appendix S1 — List of specimens examined morphologically. (PDF) [file pone.0101847.s002.pdf]

Appendix S1. Additional *Tympanocryptis tetraporophora sensu lato* specimens examined. Museum registration numbers provided, with museum acronyms: QM for Queensland Museum, NMV for Museum Victoria Melbourne, SAMA for South Australian Museum.

SAMA42705; SAMA42717; SAMA42718; SAMA42731; SAMA42732; SAMA42734; SAMA42735; SAMA42736; SAMA42737; SAMA42746; SAMA42756; SAMA42757; SAMA42786; SAMA42806; SAMA42807; SAMA42808; SAMA42809; SAMA42810; SAMA42900; SAMA42901; SAMA42934; SAMA42935; SAMA42938; SAMA44689; SAMA44692; SAMA56744; SAMA56748; SAMA55733; SAMA65424; SAMA65345; SAMA65399; SAMA65398; SAMA65355; SAMA65401; SAMA65400; SAMA590; SAMA8823; SAMA26822; SAMA26823; SAMA26834; SAMA28513; SAMA28924; SAMA41302; SAMA41304; SAMA41447; SAMA41486; SAMA41504; SAMA41505; SAMA43947; SAMA44806; SAMA46819; SAMA46923; SAMA49060; SAMA49097; SAMA49098; SAMA49123; SAMA51012; SAMA56730; SAMA60569; SAMA60894; SAMA60895; SAMA60901; SAMA60947; SAMA60981; SAMA60982; SAMA61961; SAMA62415; SAMA62417; SAMA62548; SAMA62573; SAMA62574; SAMA62583; SAMA62621; SAMA63899; SAMA64957; SAMA64963; SAMA65521; SAMA65529; SAMA65531; SAMA65556; SAMA65670; SAMA66073; SAMA66436; SAMA66549; SAMA66567; SAMA62721; SAMA62689; SAMA62717; SAMA41485; SAMA62714; SAMA11179; SAMA15603; SAMA57551; NMVD2788; NMVD41583; NMVD58315; NMVD52085; NMVD48848; NMVD48849; NMVD50988; NMVD41584; NMVD52117; NMVD52083; NMVD52086; NMVD52118; NMVD56312; NMVD48847; NMVD48846; NMVD2334; NMVD2335; NMVD5397; NMVD5398; NMVD3484; NMVD2694; NMVD3485; NMVD505; NMVD66597; NMVD74073; NMVD74074; NMVD74075; NMVD74048; NMVD74049; NMVD74050; NMVD74051; NMVD74046; NMVD41615; NMVD58492; NMVD41588; NMVD5080; NMVD728; NMVD730; NMVD16508; NMVD1119; NMVD1120; NMVD56313; NMVD3018; NMVD3036; NMVD3053; NMVD3054; NMVD2882; NMVD2883; NMVD7701; NMVD7849; NMVD69644; NMVD71433; NMVD71388; NMVD71389; NMVD71390; NMVD71391; NMVD71392; NMVD71393; NMVD71394; NMVD71355; NMVD71356; NMVD71357; NMVD71358; NMVD71359; NMVD71360; NMVD71361; NMVD71362; NMVD71363; NMVD71364; NMVD71365; NMVD71366; NMVD71367; NMVD72715; NMVD72717; NMVD72770; NMVD72754; NMVD72768; NMVD72752; NMVD72755; NMVD72756; NMVD72757; NMVD72758; NMVD72759; NMVD72760; NMVD72761; NMVD72762; NMVD72763; NMVD72764; NMVD72765; NMVD72766; NMVD72694; NMVD72739; NMVD72740; NMVD72744; NMVD72741; NMVD72736; NMVD72753; NMVD71386; NMVD71387; QMJ5817 ; QMJ5821 ; QMJ13009 ; QMJ24489 ; QMJ24490; QMJ43235; QMJ43236 ; QMJ47676 ; QMJ47677 ; QMJ47792 ; QMJ54133 ; QMJ58868; QMJ61169; QMJ74011 ; QMJ74792 ; QMJ74793; QMJ76303 ; QMJ83852 ; QMJ83853; QMJ83854; QMJ86167 ; QMJ86172 ; QMJ81203 ; QMJ84187 ; QMJ86157; QMJ63876; QMJ81784; QMJ81870; QMJ81871; QMJ82190; QMJ82191; QMJ82194; QMJ83500; QMJ83530; QMJ84184; QMJ84185; QMJ84186; QMJ87307; QMJ88329; QMJ89119; QMJ87636; QMJ34744; QMJ82087; QMJ82088; QMJ83153; QMJ8604; QMJ85233; QMJ84658; QMJ14350; QMJ65592; QMJ65593; QMJ65505; QMJ48542; QMJ65503; QMJ65504; QMJ48454; QMJ48455; QMJ48456; QMJ48457; QMJ48458; QMJ48460; QMJ48461; QMJ48462; QMJ48463; QMJ48470; QMJ48506; QMJ48508; QMJ77172; QMJ43233; QMJ86156; QMJ73051; QMJ74016; QMJ41601; QMJ32187; QMJ24826; QMJ26065; QMJ35569; QMJ26508; QMJ82149; QMJ73122; QMJ40202; QMJ78731.
